# Transcutaneous carbon dioxide improves joint inflammation and articular cartilage degeneration in rat osteoarthritis models

**DOI:** 10.1302/2046-3758.1410.BJR-2024-0338.R3

**Published:** 2025-10-22

**Authors:** Changxin Li, Hideki Moriyama, Shota Inoue, Junpei Hatakeyama, Daisuke Takamura, Hanlin Jiang, Yoshitada Sakai, Toshihiro Akisue

**Affiliations:** 1 Department of Rehabilitation Medicine, Affiliated Hospital of Zunyi Medical University, Zunyi, China; 2 Department of Rehabilitation Science, Graduate School of Health Sciences, Kobe University, Kobe, Japan; 3 Life and Medical Sciences Area, Health Sciences Discipline, Kobe University, Kobe, Japan; 4 Department of Rehabilitation, Kobe City Medical Center General Hospital, Kobe, Japan; 5 Division of Rehabilitation Medicine, Kobe University Graduate School of Medicine, Kobe, Japan

**Keywords:** Destabilization of the medial meniscus, Inflammation, Monosodium iodoacetate, Osteoarthritis, Transcutaneous carbon dioxide, degeneration of articular cartilage, Osteoarthritis (OA), carbon, rat models, knees, chondrocytes, collagen, interleukin-6, knee joints, intra-articular injection

## Abstract

**Aims:**

As global ageing and life expectancy increase, the prevalence and incidence of osteoarthritis (OA) are expected to rise. Transcutaneous carbon dioxide (CO_2_) therapy has been shown to promote muscle regeneration, fracture healing, strengthen athletic endurance, and aid recovery from peripheral nerve damage and cancer. However, its effect on symptom modification and inflammation in OA is largely unknown. This study aimed to examine whether CO_2_ therapy could slow the progression of OA and relieve OA-related inflammation in a chemically or surgically induced model in rats.

**Methods:**

OA model was induced in 32 nine-week-old male Wistar rats by intra-articular injection of monosodium iodoacetate (MIA) and surgically induced by destabilization of the medial meniscus (DMM) in the knee joint. The pathogenesis period of MIA was set at two weeks, and for DMM at four weeks. After the creation of the OA model, either CO_2_ therapy or sham intervention was applied daily for 20 minutes, and treatment was applied at two weeks. Behavioural assessments were completed at the end of the intervention period, and then knee joints were harvested. Non-demineralized frozen sections were prepared, and samples were examined histologically.

**Results:**

Assessments of knee joint diameter showed that knee swelling in the DMM model improved significantly after two weeks of CO_2_ therapy compared to the control group. The histomorphometric evaluation showed a significant increase in chondrocyte density in the CO_2_ group compared to the MIA and DMM groups. Furthermore, the number of matrix metalloproteinase 13 (MMP13), a disintegrin and metalloproteinase with thrombospondin motifs 5 (ADAMTS5), and proinflammatory cytokines tumour necrosis factor-α (TNF-α), interleukin (IL)-1β, and IL-6 positive cells decreased in the CO_2_ group. In contrast, the number of aggrecan and type II collagen-positive cells increased.

**Conclusion:**

Our results demonstrate that transcutaneous CO_2_ therapy improves OA-related inflammation and suppresses the degeneration of articular cartilage.

Cite this article: *Bone Joint Res* 2025;14(10):888–900.

## Article focus

- Transcutaneous CO_2_ therapy’s effect on symptom modification and inflammation in OA is largely unknown.
- By using chemically and surgically induced models of OA, we sought to clarify whether transcutaneous CO_2_ therapy improves OA-related inflammation and suppresses articular cartilage degeneration.

## Key messages

- Transcutaneous CO_2_ therapy reduced OA-induced articular inflammatory response and improved the imbalance between chondrocyte anabolic and catabolic processes.
- Transcutaneous CO_2_ therapy provides better pain and swelling control in post-traumatic OA.
- Transcutaneous CO_2_ therapy inhibits the development of OA by improving extracellular matrix synthesis and degradation imbalance.

## Strengths and limitations

- We first indicated that transcutaneous application of CO_2_ may have therapeutic potential for improving articular inflammation and degeneration of articular cartilage in OA patients, providing a novel strategy for treating OA.
- The male Wistar rats used in this study cannot fully reflect human variability (such as age, race, sex, genetic variation, and lifestyle). Therefore, further clinical studies are needed to translate our results to humans.

## Introduction

Osteoarthritis (OA) is a complex chronic degenerative joint disorder characterized by articular cartilage destruction, subchondral bone changes, and synovitis formation.^1-4^ The Global Burden of Disease Study System analysis reported that 300 million individuals worldwide lived with OA in 2017.^5^ OA causes pain and immense discomfort to individuals who are affected.

Aggrecan and collagen are the main components of the extracellular matrix (ECM) of cartilage. In normal joints, a dynamic equilibrium is maintained in the ECM by regulating synthesis and degradation. However, balance is disrupted during OA, leading to continual cartilage degradation. Additionally, during OA, the activation of inflammatory cytokines, such as interleukin (IL)-1β, tumour necrosis factor-α (TNF-α), and IL-6, is a key mediator in OA pathogenesis, which is closely related to cartilage matrix degradation.^6^ The current OA treatment should be aimed at relieving pain and joint stiffness, increasing joint function and mobility, and improving a patient’s quality of life by limiting the progression of damage.^7^ The treatment methods can be divided into three categories: non-drug treatment, surgical treatment, and drug treatment.^8^ Exercise and braces are mainly used to treat the knee as a non-pharmacological treatment. They can effectively increase muscle strength and reduce the body’s weight load, but fail to address the evolving and complex nature of OA. Commonly prescribed analgesics and non-steroidal anti-inflammatory drugs (NSAIDs) only provide symptomatic relief, and the long-term use of these drugs may induce prominent side effects. Joint arthroplasty surgery may be recommended in late knee OA, where most articular cartilage has been worn out. Joint arthroplasty can eliminate knee pain, readjust the joints, and greatly restore joint function to the knee. However, surgery is often used as a last resort after the failure of drug treatment; therefore, patients usually experience debilitating pain for many years. Taken together, our goal is to develop non-invasive treatment regimens to relieve pain and treat inflammation.

The therapeutic effect of transcutaneous carbon dioxide (CO_2_) therapy is based on the Bohr effect, caused by an increase in blood flow and microcirculation, and a partial increase in O_2_ pressure in local tissues.^9,10^ This has been used to treat cardiac disease and skin problems.^11^ Additionally, in animal studies, CO_2_ therapy accelerates the performance of endurance exercise,^12^ prevents muscle atrophy,^13-15^ promotes fracture healing,^16,17^ and inhibits muscle atrophy after fracture.^18^ Furthermore, it has ameliorated relatively deep knee joint contractures.^19,20^ Here, joint contractures could be improved by improving synovial fibrosis associated with spinal cord injury or joint immobilization. These findings support the possibility that CO_2_ therapy may be effective for OA occurring in the deeper layers of the body.

Some CO_2_ therapeutic methods have been reported in recent decades; however, to the best of our knowledge, the effects of CO_2_ therapy on inflammation in OA have not been studied. We investigated the regulation of inflammatory processes and OA progression by CO_2_ therapy in monosodium iodoacetate (MIA)- and destabilization of the medial meniscus (DMM)-induced OA models.

## Methods

### Experimental design and animal care

In total, 32 male Wistar rats (Japan SLC Inc., Japan) aged nine weeks were used for this study. Male animals were selected to avoid the effects of oestrogen on bone metabolism.^21^ Acclimated for one week, these rats were equally divided into two experimental protocols: MIA and DMM. The animals in each experimental protocol were divided randomly into four subgroups (n = 4 per group): control, saline injection (sham), untreated after MIA (MIA), and transcutaneous CO_2_ therapy treated after MIA (CO_2_) for the MIA protocol; and control, sham operation (sham), DMM, and transcutaneous CO_2_ treated after DMM (CO_2_) for the DMM protocol. The DMM surgery was performed on the right knee, while the left knee was the contralateral control for comparison as previously described.^19^ Two weeks of the CO_2_ treatment was started after two or four weeks of MIA injection or DMM surgery, respectively. The subgroup sample sizes were calculated by a power analysis based on pilot results for detecting a 10° difference in ROM 19 times out of 20. Animals were fed ad libitum and kept in a thermostatic environment at 21°C under a 12-hour light/12-hour dark cycle. We have adhered to the ARRIVE guidelines and have included the ARRIVE checklist in the Supplementary Material. This study was conducted following the Regulations for the Conduct of Animal Experiments and was approved by the Animal Experimentation Committee of Kobe University (approval number: P180704).

### OA models

For the chemically induced model of OA, intra-articular injection of MIA inhibits glyceraldehyde-3-phosphate dehydrogenase activity in chondrocytes, disrupting cellular glycolysis and eventually resulting in chondrocyte death.^22,23^ MIA is the most often used in all chemical models of OA, as intra-articular injection of MIA in rodents reproduces OA-like lesions and functional impairment that can be analyzed and quantified.^24,25^ In the pathological models for OA, injection of MIA into the rat femorotibial joint space has produced a linear pathology similar to OA.^26^ Compared with other experimental models, the MIA-induced OA model is highly reproducible and mimics OA in humans.^27^ According to a previous study,^28^ for induction of MIA-induced arthritis, rats were anaesthetized with NARCOBIT-EII type (Natsume Seisakusyo Inc., Japan), and a volume of 25 μl sterile saline solution with 3 mg MIA (I2512; Sigma-Aldrich, USA) was injected into the knee joint through the right infrapatellar ligament. The sham group received an equal volume of 0.9% sterile saline injected into the right knee joint through the right infrapatellar ligament. The contralateral left knee did not receive treatment. MIA was prepared in sterile conditions and injected using a Hamilton syringe and dispenser (PB600-1; Hamilton Company, USA) with a 27-gauge needle inserted into the joint. These rats were subcutaneously administered buprenorphine 0.05 mg/kg as the analgesic every 12 hours for three days after injection to reduce postoperative pain as much as possible. MIA injection may cause a systemic inflammatory response, so the right contralateral knee did not receive any treatment.

The DMM model induces OA with great ease and reproducibility, and has been widely used in basic research on common knee OA.^29^ It has become the gold standard in post-traumatic OA. The DMM-induced OA model was created on the right knee as previously described.^29^ Briefly, after the rats were anaesthetized by isoflurane with NARCOBIT-EII type, the right knee joint was exposed following a medial capsular incision and gentle lateral displacement of the knee extensor muscles without transection of the patellar ligament. Then, the medial meniscotibial ligament was transected, leading to DMM. A sham operation received the same approach without the medial meniscotibial ligament transection. These rats were subcutaneously administered buprenorphine 0.05 mg/kg as the analgesic every 12 hours for three days after surgery to reduce the postoperative pain as much as possible.

### Transcutaneous CO_2_ therapy

Animals were anaesthetized with isoflurane (095-06573; FUJIFILM Wako Pure Chemical Corporation, Japan), and a transcutaneous CO_2_ absorption-enhancing hydrogel (NeoChemir Inc., Japan) was applied to the hind limbs of rats as previously described.^14^ Briefly, we shaved the hair on the lower limbs, and the hydrogel was used, which promoted the absorption of the CO_2_ to the entire knee joint. The CO_2_ adaptor was attached to the limbs and sealed; diluted 100% CO_2_ gas was exposed and percutaneously absorbed into the whole hind limb. Each treatment was applied for 20 mins/day, seven days/week, for two weeks. Sham animals in this study were divided into two groups: surgical sham and hydrogel sham. The surgical sham group underwent the same surgical procedure without MIA/DMM induction, serving as a control for the surgical intervention itself. The hydrogel sham group received hydrogel treatment with ambient air instead of CO_₂_, ensuring that any effects observed were due to CO_₂_ exposure rather than the hydrogel application. The CO_2_ gas flowed in the CO_2_ adaptor to absorb it sufficiently, and the hair on the lower limb was shaved once a week. All animals survived throughout the experimental period and appeared to be healthy.

### Behavioural testing

Mechanical stimulation was assessed by measuring withdrawal thresholds to calibrated von Frey filaments as previously described.^30^ The 50% withdrawal threshold was determined using the up-down method. Nine hairs ranging from 0.04 to 15 g were used. Testing was initiated with the 2.0 g hair in the middle of the series. Stimuli were presented at intervals of several seconds, allowing for the apparent resolution of any behavioural responses to previous stimuli. A positive response was noted if the paw was sharply withdrawn. Flinching immediately upon removal of the hair was also considered a positive response. Ambulation was considered an ambiguous response, and the stimulus was repeated in such cases. If the withdrawal threshold did not match the previous testing session in a given hind paw, the next largest hair in the series was tested. Accordingly, although all responses were noted, counting of the critical six data points did not begin until the response threshold was first crossed, at which time the two responses straddling the threshold were retrospectively designated as the first two responses of the six series. Four additional responses to the continued presentation of stimuli that were varied sequentially up or down, based on the rat’s response, constituted the remainder of the series. The rats were assessed just before the injection and at points 1, 4, 7, and 14 days after the MIA intra-articular injection (for the DMM group, at points 1, 4, 7, 14, 21, and 28 days) and at points 1, 4, 7, and 14 days after the transcutaneous CO_2_ intervention.

### Joint swelling score

The joint swelling scores were measured under isoflurane anaesthesia in rats. Then, knee width diameters were measured to infer joint swelling as an indicator of inflammation resulting from MIA injection or DMM operation. The width of the knee joint was measured with a manual caliper before the injection and at points 1, 4, 7, and 14 days after the MIA intra-articular injection (for the DMM group, at points 1, 4, 7, 14, 21, and 28 days) and at points 1, 4, 7, and 14 days after the transcutaneous CO_2_ intervention. Two blinded raters (CL, SI) scored knee joint swelling in both groups of rats.

### Histology

At the end of the experimental period, all animals were euthanized by exsanguination under anaesthesia and analgesia. We prepared undecalcified frozen sections according to the method described by Kawamoto.^31^ Briefly, the whole knee joints, including the patella and joint capsule, were harvested. Knee samples were then freeze-embedded with 5% carboxymethyl cellulose gel and subsequently stored in a freezer at −75°C. Blocks were cut into slices from the medial side of the knees, and 5 μm sagittal sections were prepared at the level of 50 to 200 μm lateral from the level at the medial meniscus, dividing into anterior and posterior horns. Four samples from each group were used for histological, histomorphometric, or immunohistochemical analyses.

### OARSI score

Cartilage degradation of OA in all samples was assessed using the semiquantitative Osteoarthritis Research Society International (OARSI) scoring system for medical femoral condyle and medial tibial plateau.^32,33^ Then, a summed OARSI score of the medial femoral condyle and the medial tibial plateau was used to evaluate the degree of articular cartilage destruction in a blinded manner. Three blinded assessors (CL, SI, JH) scored two regions (medial tibial plateaus and medial femoral condyles) of the knee from four rats per group. Higher scores indicate greater severity of symptoms (grade 0 to 6).

### Synovitis score

Knee sagittal sections were stained with haematoxylin and eosin (H&E). The synovial insertion of the lateral femur, medial femur, lateral tibia, and medial tibia were evaluated separately with a modified form of an established synovitis score for changes in synovial lining thickness and cellular density in the synovial stroma (maximum site score 6).^34^

### Histomorphometric analysis

According to the method described by Nomura et al,^35^ the posterior femur and middle tibial were defined as the regions of the articular cartilage evaluation area. Articular cartilage thickness and chondrocyte density were measured with digitized images of histological sections stained with H&E. For each region, a 200 μm-long stretch of the cartilage surface was defined, and the uncalcified and calcified cartilage under this stretch was measured separately. The thickness of each layer was calculated by dividing the area by 200 μm. Total cartilage thickness was the sum of the thickness of the uncalcified and calcified layers. The mean thickness for each specimen was derived by averaging measurements from three slides spaced 50 μm apart. The cartilage area was measured using the same method as cartilage thickness, and cells with visible nuclei in the area were counted manually. Chondrocyte density was determined as the number of chondrocytes per cartilage area.

### Immunohistochemistry

According to the protocols established by Moriyama et al,^36^ the frozen sections were incubated with anti- IL-6 (diluted 1:200, AB6672; Abcam, UK), anti-TNF-α (diluted 1:100, ab6671; Abcam), anti-type II collagen (diluted 1:200, ab21291; Abcam), anti-matrix metallopeptidase 13 (MMP13) (diluted 1:400, ab39012; Abcam), anti-aggrecan (diluted 1:400, AB1031; Millipore, USA), and anti-a disintegrin and metalloproteinase with thrombospondin motifs 5 (ADAMTS5; diluted 1:500, ab41037; Abcam) antibodies. A subsequent reaction was made using the streptavidin-biotin-peroxidase complex technique with an Elite ABC kit (diluted 1:50, PK-610; Vector Laboratories, USA). Then, the ImmPACT DAB (SK-4105; Vector Laboratories) was used for the visualization of the immunoreaction. The sections were finally counterstained with haematoxylin, washed in water, and coverslipped. They were captured with a light microscope (BX-53; Olympus, Japan) at a magnification of ×4 or ×20 and acquired as digital images.

### Statistical analysis

Statistical analyses were conducted with EZR (Saitama Medical Center, Jichi Medical University; Japan), which is a graphical user interface for R (The R Foundation for Statistical Computing, Austria).^37^ First, the Shapiro-Wilk test was used to check for the normality of the distribution for each data set. Data from behavioural tests and knee oedema measured over multiple timepoints were analyzed using repeated-measures analysis of variance (ANOVA). The OARSI score, synovium inflammation score, and results of the behavioural testing were compared among groups using the Kruskal-Wallis test with a Steel-Dwass post hoc analysis. The results of knee oedema and histomorphological and immunohistochemical analysis were compared among groups using analysis of variance with Tukey’s post hoc test. Parametric data are shown as the mean (SD), whereas nonparametric data are shown as the median (IQR). All significance thresholds were set at 5%. A post hoc power analysis was performed using G*Power 3 programme^38^ to confirm that sufficient numbers of animals were used.

## Results

### Effects of transcutaneous **CO_2_** therapy on pain‐related behaviour and joint swelling

We assessed mechanical hypersensitivity using pain behaviour tests (von Frey filaments) at 0, 1, 4, 7, 14, 15, 18, 21, and 28 days after the MIA injection (Table I) or 0, 1, 4, 7, 14, 21, 28, 29, 32, 35, and 42 days after DMM surgery (Table II). The 50% paw withdrawal threshold tended to decrease in the MIA group at day 7 to 18 and in the DMM group at day 4 to 14 after OA induction compared to that in the sham group, but the differences were not significant (MIA vs sham group: 7d, p = 0.093; 14d, p = 0.090; 15d, p = 0.084; 18d, p = 0.093; DMM versus sham group: 4d, p = 0.084; 7d, p = 0.093; 14d, p = 0.093; Power = 0.80; repeated-measures ANOVA and Kruskal-Wallis test followed by Steel-Dwass post hoc tests). The CO_2_ therapy tended to improve pain behaviour at 42 days after the DMM surgery compared to the DMM group (p = 0.063, Power = 0.80; repeated-measures ANOVA and Kruskal-Wallis test followed by Steel-Dwass post hoc tests), whereas there was no significant difference in the other comparisons. Next, to investigate the development of the joint inflammatory swelling, we assessed the knee width (Table I and Table II). The knee width significantly increased in the MIA group from day 1 to 7 (MIA versus control group: 1d, p < 0.001; 4d, p < 0.001; 7d, p < 0.001; MIA versus sham group: 1d, p = 0.001; 4d, p < 0.001; 7d, p = 0.003; Power = 0.80; repeated-measures ANOVA and one-way ANOVA with post hoc Tukey tests) and in the DMM group from day 1 to the end of the experiment on day 42 (DMM versus control group: 1d, p < 0.001; 4d, p < 0.001; 7d, p < 0.001; 14d, p < 0.001; 21d, p < 0.001; 28d, p < 0.001; 29d, p < 0.001; 32d, p < 0.001; 35d, p < 0.001; 42d, p < 0.001; DMM vs sham group: 1d, p < 0.001; 4d, p < 0.001; 7d, p = 0.002; 14d, p < 0.001; 21d, p = 0.004; 28d, p < 0.001; 29d, p < 0.001; 32d, p = 0.002; 35d, p < 0.001; 42d, p < 0.001; Power = 0.80; repeated-measures ANOVA and one-way ANOVA with post hoc Tukey tests) after OA induction compared with rats in the control and sham groups. The CO_2_ therapy decreased the DMM-induced knee width at days 29 and 42 (DMM versus CO_2_ group: 29d, p = 0.016; 42d, p = 0.029; Power = 0.80; repeated-measures ANOVA and one-way ANOVA with post hoc Tukey tests), but there were no differences between the MIA and MIA + CO_2_ groups at any timepoint.

**Table I.** Development of mechanical allodynia and knee oedema in the monosodium iodoacetate (MIA) model.

Measurement	Group	Days								
		0	1	4	7	14	15	18	21	28
Median 50% paw withdrawal threshold, g (IQR)	Control	6.54 (5.98 to 7.10)	6.29 (5.94 to 7.10)	6.45 (6.10 to 7.10)	5.73 (5.63 to 6.09)	6.90 (6.90 to 7.10)	6.37 (5.98 to 6.58)	6.27 (5.86 to 7.10)	6.49 (6.29 to 6.49)	6.90 (6.90 to 7.10)
	Sham	7.10 (7.10 to 7.78)	3.96 (2.97 to 4.76)	3.07 (2.36 to 3.37)	3.65 (3.26 to 4.09)	4.04 (3.78 to 4.57)	3.07 (2.97 to 3.07)	4.98 (4.27 to 5.79)	4.04 (3.78 to 4.57)	3.37 (2.97 to 3.37)
	MIA	5.86 (5.13 to 6.58)	2.42 (2.06 to 2.69)	1.87 (1.40 to 2.10)	1.56 (1.41 to 1.60)	1.36 (1.16 to 1.47)	1.53 (1.26 to 1.87)	2.73 (2.54 to 2.97)	2.97 (2.97 to 2.97)	3.08 (2.97 to 3.08)
	CO_2_	6.75 (6.37 to 7.10)	2.73 (2.54 to 2.97)	1.71 (1.71 to 1.80)	1.27 (1.06 to 1.50)	1.37 (0.83 to 1.67)	1.81 (1.39 to 1.93)	2.74 (2.74 to 2.97)	2.69 (2.29 to 3.07)	2.84 (2.74 to 3.07)
Mean transverse diameter, mm (SD)	Control	7.81 (0.14)	7.77 (0.05)	7.89 (0.00)	7.90 (0.03)	7.84 (0.04)	7.82 (0.04)	7.72 (0.02)	7.86 (0.02)	7.82 (0.04)
	Sham	7.69 (0.06)	8.19 (0.27)	7.95 (0.13)	8.09 (0.14)	8.05 (0.08)	8.09 (0.10)	8.02 (0.11)	8.03 (0.02)	7.98 (0.04)
	MIA	7.74 (0.05)	9.64 (0.25)*†	9.12 (0.12)*†	8.73 (0.03)*†	8.49 (0.06)*	8.59 (0.11)*	8.49 (0.08)*	8.26 (0.10)	8.32 (0.09)
	CO_2_	7.77 (0.01)	9.03 (0.10)*†	8.5 (0.20)*†‡	8.36 (0.13)*	8.14 (0.18)	8.21 (0.20)	8.13 (0.20)	8.17 (0.22)	8.03 (0.23)

* p < 0.05 vs control (the p-values for knee oedema were analyzed by repeated-measures analysis of variance (ANOVA) or one-way ANOVA followed by Tukey's post hoc test, while the p-values for mechanical allodynia were analyzed by repeated-measures ANOVA or the Kruskal-Wallis test followed by the Steel-Dwass post hoc analysis).

† p < 0.05 vs sham.

‡ p < 0.05 vs MIA.

**Table II.** Development of mechanical allodynia and knee oedema in the destabilization of the medial meniscus (DMM) model.

Measurement	Group	Days										
		0	1	4	7	14	21	28	29	32	35	42
Median 50% paw withdrawal threshold, g (IQR)	Control	7.10 (6.82 to 9.08)	6.54 (5.98 to 7.10)	6.34 (5.78 to 7.10)	5.98 (5.13 to 6.50)	5.73 (5.29 to 5.77)	5.93 (5.32 to 6.26)	6.09 (5.86 to 6.58)	5.19 (4.57 to 5.56)	5.58 (5.13 to 5.77)	5.38 (4.57 to 6.09)	5.66 (4.57 to 6.58)
	Sham	8.08 (6.66 to 9.06)	3.45 (2.21 to 4.76)	3.37 (2.97 to 3.37)	4.57 (4.17 to 5.27)	2.91 (2.25 to 3.28)	4.40 (2.97 to 5.20)	3.55 (2.97 to 4.09)	3.56 (2.97 to 3.56)	4.08 (3.90 to 4.57)	2.97 (2.97 to 2.97)	3.37 (2.97 to 3.37)
	DMM	7.10 (6.47 to 7.78)	2.26 (1.61 to 2.97)	1.90 (1.68 to 2.08)	1.89 (1.45 to 2.27)	1.12 (0.91 to 1.44)	2.17 (1.42 to 2.97)	3.11 (2.84 to 3.24)	2.45 (2.00 to 2.97)	3.01 (2.74 to 3.43)	2.97 (2.97 to 2.97)	2.97 (2.97 to 2.97)
	CO_2_	7.10 (7.10 to 7.81)	3.22 (2.82 to 3.37)	2.53 (2.22 to 2.97)	2.52 (2.06 to 2.97)	1.32 (1.23 to 1.53)	1.98 (1.72 to 2.20)	2.36 (2.00 to 2.69)	2.30 (2.00 to 2.51)	3.22 (2.82 to 3.37)	4.32 (2.97 to 5.31)	4.81 (4.35 to 5.03)
Mean transverse diameter, mm (SD)	Control	7.67 (0.04)	7.76 (0.03)	7.67 (0.01)	7.68 (0.10)	7.90 (0.05)	8.00 (0.03)	7.91 (0.04)	7.90 (0.03)	7.84 (0.02)	8.00 (0.02)	7.96 (0.03)
	Sham	7.58 (0.07)	8.10 (0.20)	8.32 (0.13)*	8.47 (0.06)*	8.56 (0.05)*	8.30 (0.05)	8.24 (0.08)	8.18 (0.09)	8.24 (0.06)	8.27 (0.10)	8.16 (0.13)
	DMM	7.58 (0.04)	9.82 (0.17)*†	9.74 (0.10)*†	9.48 (0.19)*†	9.62 (0.12)*†	8.93 (0.17)*†	9.21 (0.12)*†	9.29 (0.07)*†	9.08 (0.22)*†	9.08 (0.11)*†	9.16 (0.12)*†
	CO_2_	7.51 (0.07)	9.65 (0.18)*†	9.41 (0.15)*†	8.93 (0.21)*	9.07 (0.20)*‡	8.78 (0.09)*†	8.77 (0.14)*†‡	8.85 (0.12)*†‡	8.73 (0.10)*	8.83 (0.09)*†	8.71 (0.08)*†‡

* p < 0.05 vs control (the p-values for knee oedema were analyzed by repeated-measures analysis of variance (ANOVA) or one-way ANOVA followed by Tukey's post hoc test, while the p-values for mechanical allodynia were analyzed by repeated-measures ANOVA or the Kruskal-Wallis test followed by the Steel-Dwass post hoc analysis).

† p < 0.05 vs sham.

‡ p < 0.05 vs DMM.

### Transcutaneous **CO_2_** therapy has no effect on OARSI and synovitis score

**Fig. 1:**
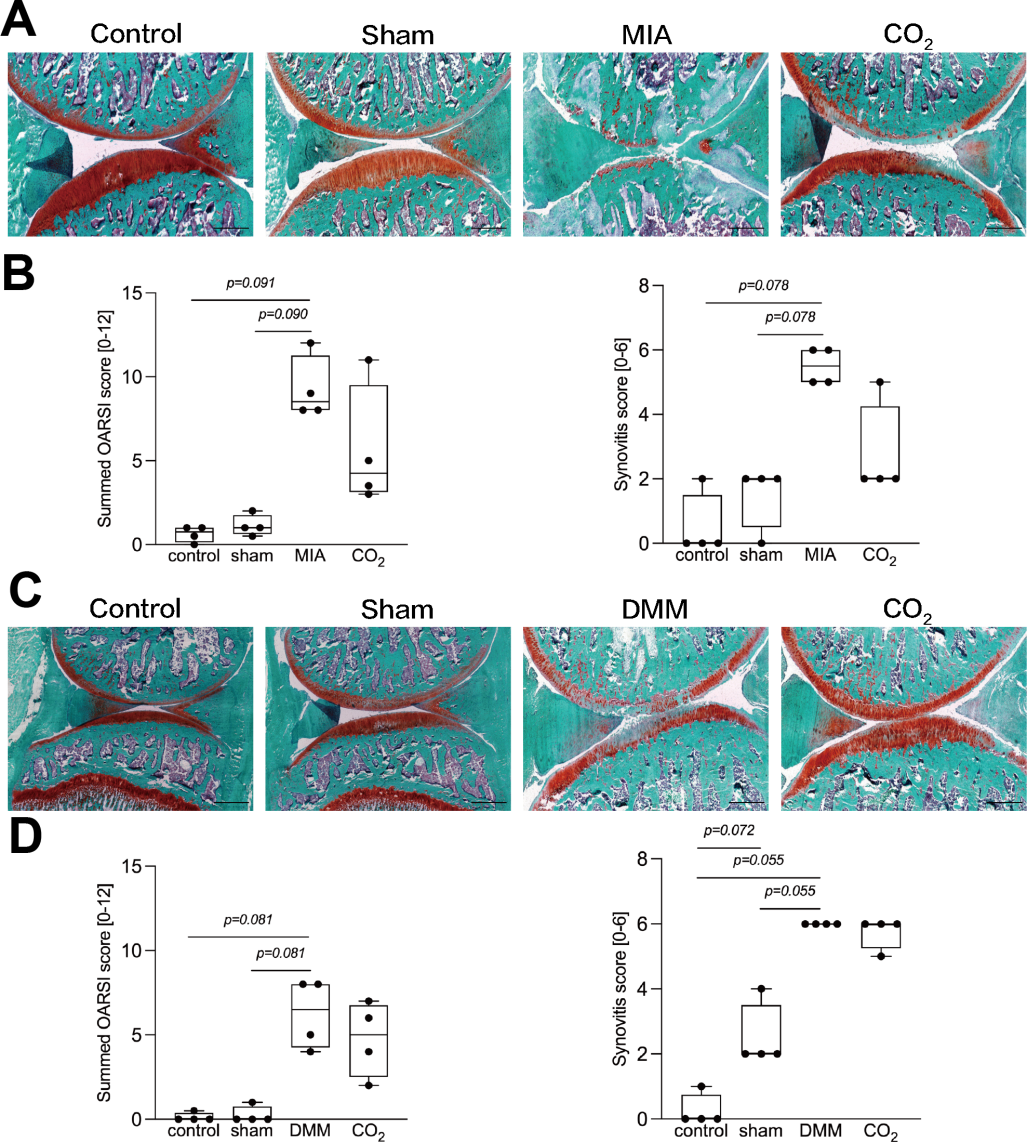
Histological changes in the monosodium iodoacetate (MIA)- and destabilization of the medial meniscus (DMM)-induced rat. a) and c) Representative photographs of Safranin O stained sagittal sections of the joints of MIA- or DMM-induced rat. Scale bar = 200 μm. b) and d) Osteoarthritis Research Society International (OARSI) and synovitis scoring of the joints of MIA- or DMM-induced rat after two weeks of transcutaneous CO_2_ application. The OARSI and synovitis scores were analyzed using the Kruskal-Wallis test followed by the Steel-Dwass post hoc test. Boxplots displaying median values and IQR (n = 4 per group).

We evaluated the cartilage degradation through OARSI scores. Although the scores appeared elevated in the MIA and DMM groups compared to the sham group, the differences were not statistically significant (MIA: p = 0.090; DMM: p = 0.081; Power = 0.80) (Figure 1). The CO_2_ group showed no significant differences compared with the DMM and MIA groups. We also evaluated the levels of synovitis with histological analysis. Although synovitis scores were higher in the MIA and DMM groups than in the sham group (p = 0.078 and p = 0.055, respectively; both Power = 0.80; Kruskal-Wallis test followed by Steel-Dwass test), these differences were not statistically significant. The synovitis score was comparable between the CO_2_ group and the DMM and MIA groups (Figure 1).

### Transcutaneous **CO_2_** therapy increases chondrocyte density but not cartilage thickness

**Fig. 2:**
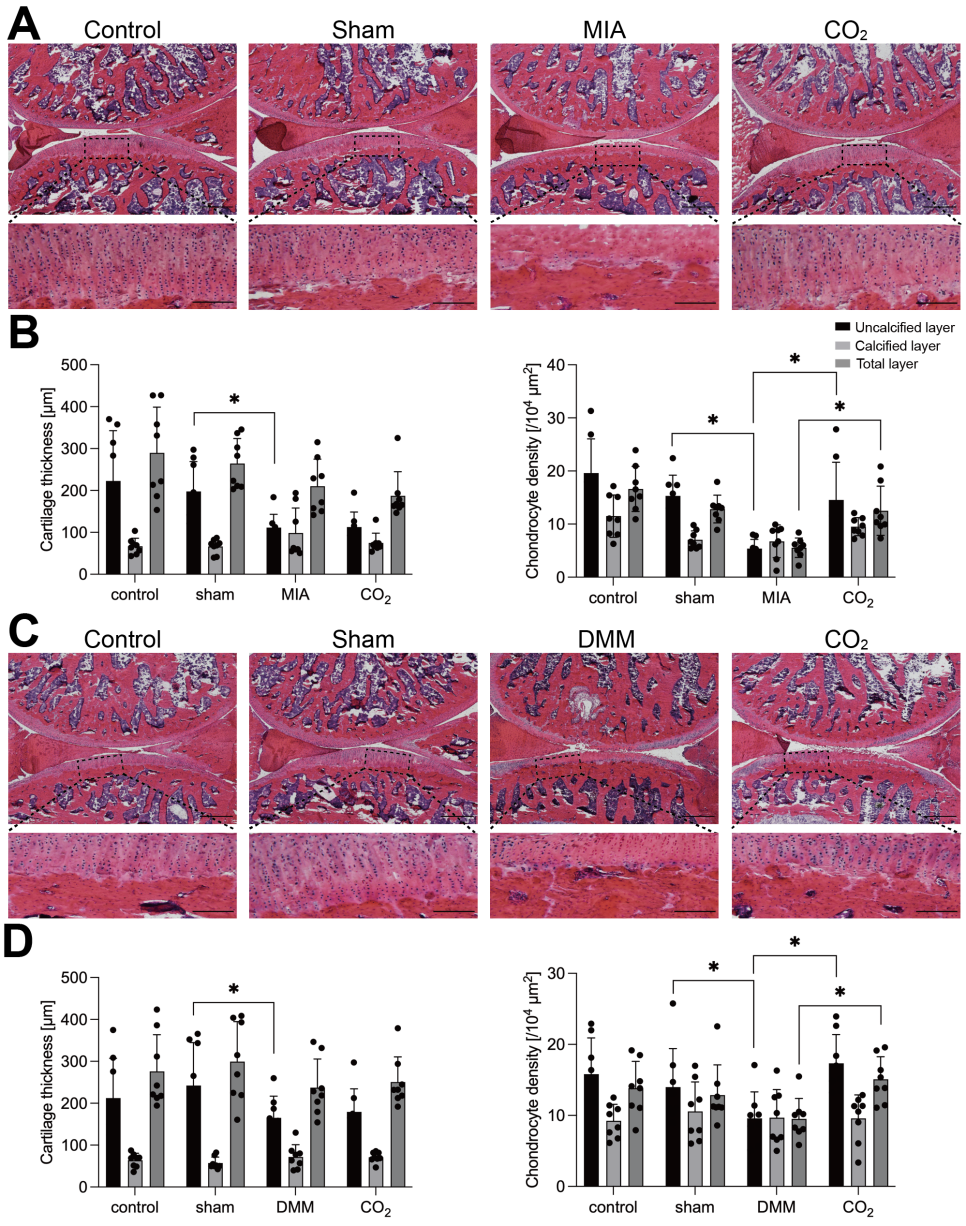
Cartilage thickness and chondrocyte density. a) and c) Representative photographs of haematoxylin and eosin (H&E) staining sagittal sections of the joints of monosodium iodoacetate (MIA)- and destabilization of the medial meniscus (DMM)-induced rat. Scale bar = 200 μm. b) and d) Thickness and density in uncalcified and calcified layer of articular cartilage were measured separately on the histological sections stained with H&E. Mean cartilage thickness and chondrocyte density of the two regions. Cartilage region: posterior femur and middle tibial. (n = 4 per group). The results were analyzed using the Shapiro-Wilk normality test and one-way analysis of variance (ANOVA) followed by Tukey's post hoc test. *p < 0.05, **p < 0.01, ***p < 0.001.

Cartilage thickness and chondrocyte density showed a significant decrease in the uncalcified layer of the MIA and DMM groups compared to the sham group, except chondrocyte density in the DMM group (Figure 2) (cartilage thickness: p = 0.001 (MIA), p = 0.037 (DMM); chondrocyte density: p = 0.008 (MIA), p = 0.087 (DMM); Power = 0.80). The chondrocyte density of the uncalcified and total layer in the CO_2_ group significantly increased compared to that in the MIA and DMM groups (Figure 2) (p = 0.014 (uncalcified MIA), p = 0.011 (total layer MIA), p = 0.003 (uncalcified DMM), p = 0.012 (total layer DMM); Power = 0.80), but there were no differences in cartilage thickness between treated and non-treated groups. The results were analyzed using the Shapiro-Wilk normality test and one-way ANOVA followed by Tukey’s post hoc test.

### Transcutaneous **CO_2_** therapy reduced matrix degradation markers in cartilage

**Fig. 3:**
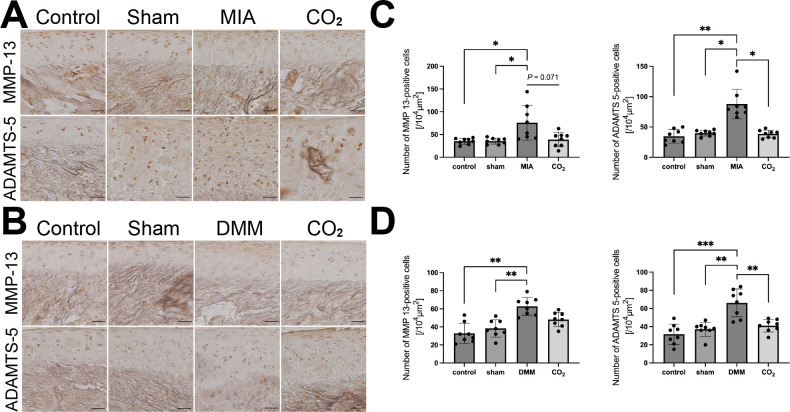
Immunohistochemical staining of a disintegrin and metalloproteinase with thrombospondin motifs 5 (ADAMTS-5) and matrix metalloproteinase 13 (MMP-13). a) and b) Representative photographs of immunohistochemical staining of ADAMTS-5 and MMP-13 of monosodium iodoacetate (MIA)- and destabilization of the medial meniscus (DMM)-induced rat after two weeks of transcutaneous CO_2_ application. Scale bar = 100 μm. c) and d) The numbers of ADAMTS-5 and MMP-13-positive cells. (n = 4 per group). The results were analyzed using the Shapiro-Wilk normality test and one-way analysis of variance (ANOVA) followed by Tukey's post hoc test. *p < 0.05, **p < 0.01, ***p < 0.001).

The levels of MMP13 and ADAMTS5 proteins in the articular cartilage were immunohistochemically evaluated (Figures 3a and 3b). The MIA and DMM groups increased MMP13 and ADAMTS5 positive cells in the cartilage compared with the sham groups (MIA, p < 0.05; DMM, p < 0.001; both Power = 0.80) (Figures 3c and 3d). The CO_2_ therapy tended to decrease MMP13 positive cells in the MIA group (p = 0.071, Power = 0.80), but not in the DMM rats. ADAMTS5 positive cells in the CO_2_ group were lower than those in the MIA and DMM groups (p = 0.039 and p = 0.008, respectively; both Power = 0.80; Shapiro-Wilk normality test and one-way ANOVA followed by Tukey’s post hoc test).

### Transcutaneous **CO_2_** therapy increased matrix synthesis markers in cartilage

**Fig. 4:**
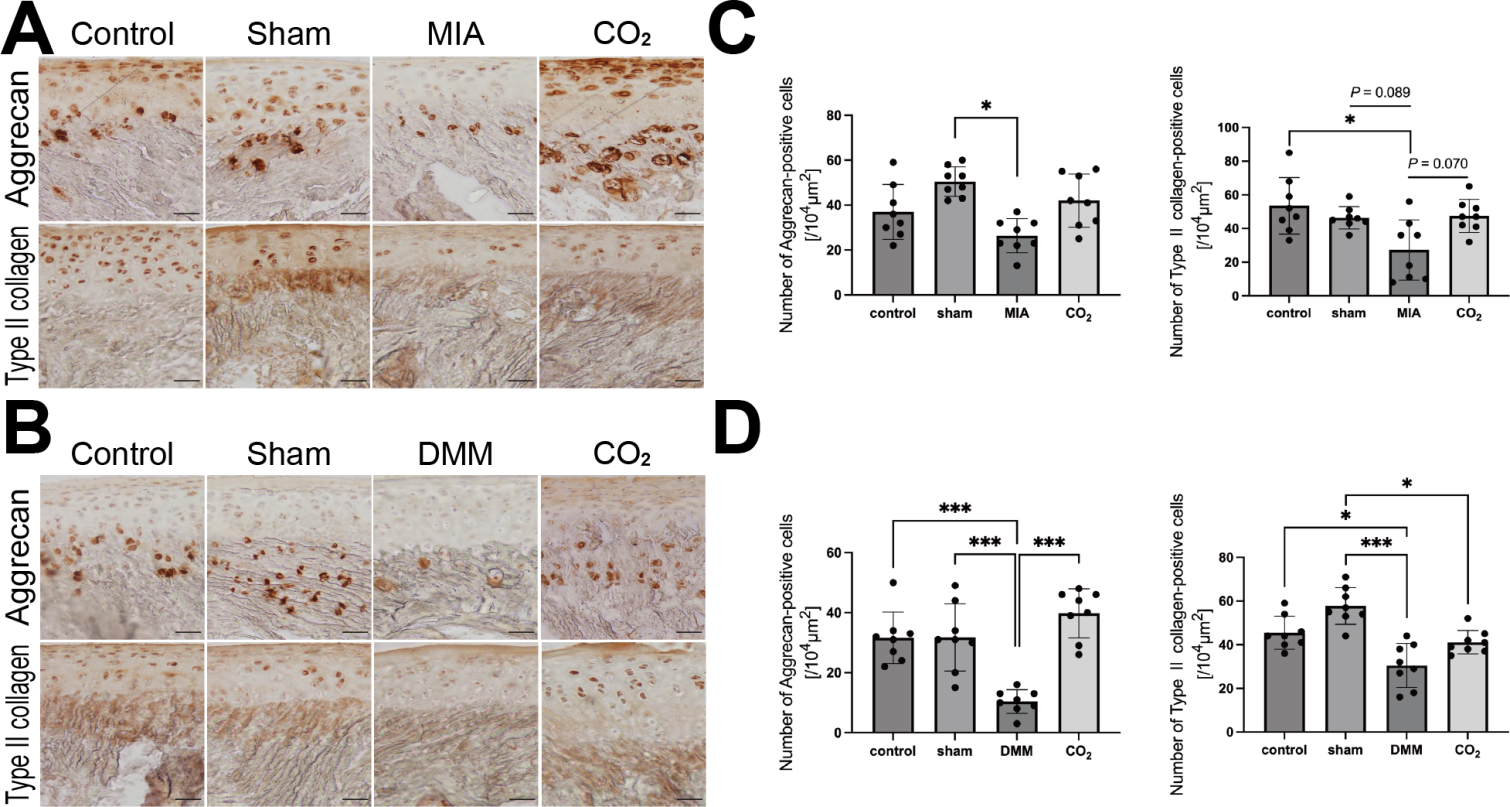
Immunohistochemical staining of type II collagen and aggrecan. a) and b) Representative photographs of immunohistochemical staining of type II collagen and aggrecan of monosodium iodoacetate (MIA)- and destabilization of the medial meniscus (DMM)-induced rat after two weeks of transcutaneous CO_2_ application. Scale bar = 100 μm. c) and d) The numbers of type II collagen and aggrecan-positive cells. (n = 4 per group). The results were analyzed using the Shapiro-Wilk normality test and one-way analysis of variance (ANOVA) followed by Tukey's post hoc test. *p < 0.05, **p < 0.01, ***p < 0.001.

The positive cells of aggrecan were decreased in the MIA and DMM groups compared with sham groups (MIA, p = 0.016; DMM, p = 0.001; both Power = 0.80) (Figure 4). Type II collagen-positive cells showed a non-significant decrease in the MIA rats (p = 0.089, Power = 0.80) and a significant decrease in the DMM rats compared to the sham rats (p = 0.001, Power = 0.80). The CO_2_ therapy tended to increase type II collagen-positive cells compared to the MIA group (p = 0.070, Power = 0.80) (Figure 4c). The number of aggrecan-positive cells in the DMM + CO_2_ group was significantly higher than that in the DMM group (p < 0.001, Power = 0.80; Shapiro-Wilk normality test and one-way ANOVA followed by Tukey’s post hoc test) (Figure 4d).

### Transcutaneous CO_2_ suppresses the expression levels of IL-6 and TNF-α inflammatory factors in articular cartilage

**Fig. 5:**
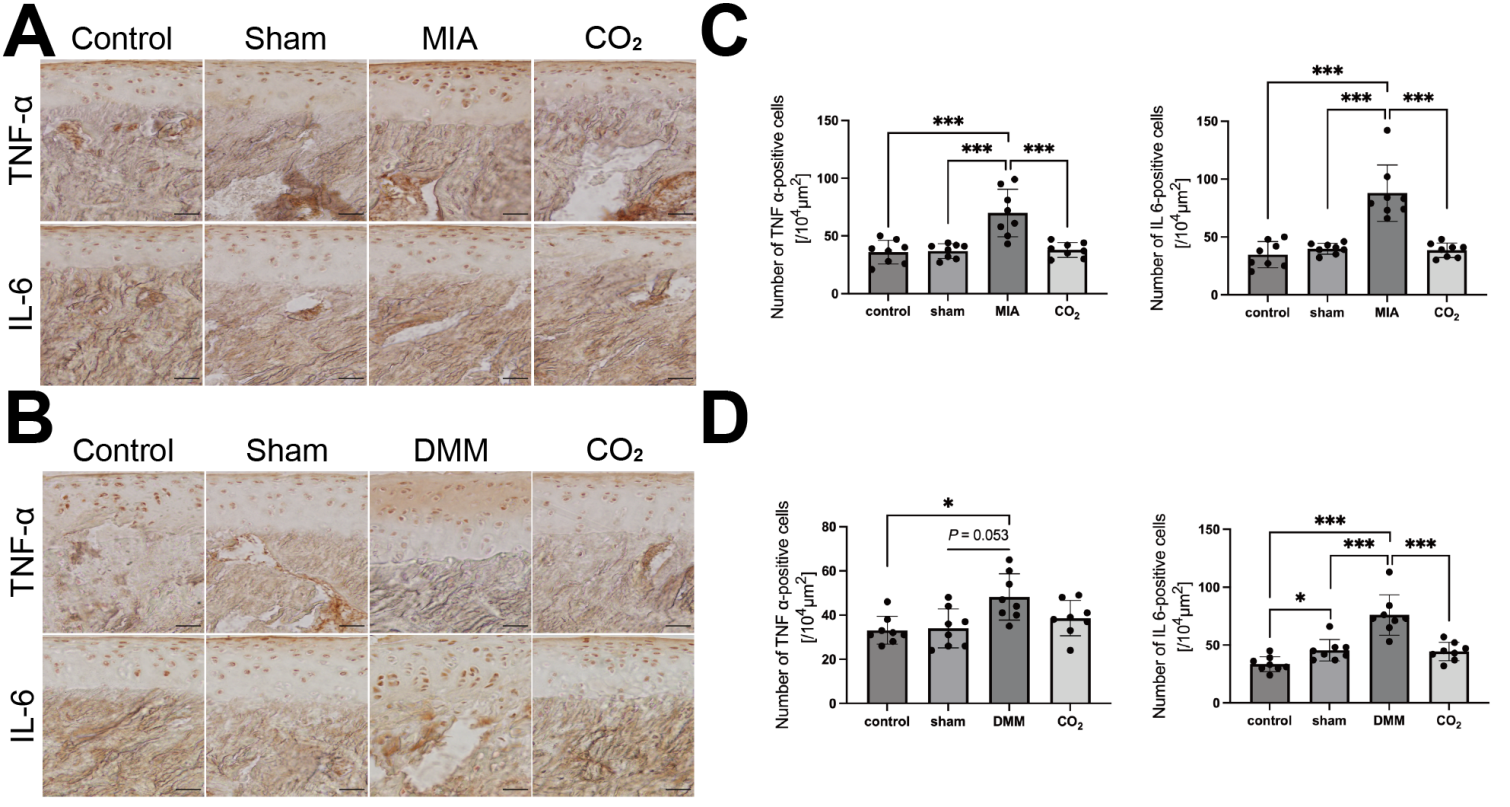
Immunohistochemical staining of interleukin-6 (IL-6) and tumour necrosis factor-α (TNF-α). a) and b) Representative photographs of immunohistochemical staining of IL-6 and TNF-α of monosodium iodoacetate (MIA)- and destabilization of the medial meniscus (DMM)-induced rat after two weeks of transcutaneous CO_2_ application. Scale bar = 100 μm. c) and d) The numbers of IL-6 and TNF-α-positive cells. (n = 4 per group). The results were analyzed using the Shapiro-Wilk normality test and one-way analysis of variance (ANOVA) followed by Tukey's post hoc test. *p < 0.05, **p < 0.01, ***p < 0.001.

To determine the inflammatory response in OA, we evaluated the TNF-α and IL-6 protein levels (Figures 5a and 5b). The MIA injection induced significant increases in TNF-α and IL-6 positive cells compared with sham groups (both p < 0.001, Power = 0.80) (Figure 5c). The DMM surgery increased TNF-α and IL-6 positive cells compared to the sham group (p = 0.053 and p < 0.001, respectively; both Power = 0.80) (Figure 5d). The CO_2_ therapy significantly decreased the number of TNF-α and IL-6 positive cells compared to the MIA and DMM groups (both p < 0.001, Power = 0.80; Shapiro-Wilk normality test and one-way ANOVA followed by Tukey’s post hoc test), except for the TNF-α positive cells in the rats which received the CO_2_ therapy after the DMM surgery.

## Discussion

OA, a chronic disease involving the degeneration of articular cartilage, is a common cause of severe disability.^39^ Transcutaneous CO_2_ therapy has been developed to prevent and treat musculoskeletal disorders in human and animal models.^13-15^ Nevertheless, the effects of CO_2_ therapy on OA joints remain unknown. This study demonstrated that CO_2_ therapy reduced pain and knee oedema in DMM-induced OA rat models. The therapy reduced knee swelling and pain-like behaviour in the DMM model, but had limited effects on the MIA model, potentially reflecting differences in the mechanisms driving pain in chemically and surgically induced OA.

Generally, the progression of OA is accompanied by secondary clinical symptoms such as, most prominently, pain and joint swelling. In the current study, we used the von Frey filament, a common method of pain assessment.^40,41^ Consistent with previous studies,^42,43^ the rats receiving MIA injection or DMM surgery tended to increase pain and joint swelling in this study, suggesting that our OA model was successfully established. However, there was no difference in the pain and joint swelling observed in the CO_2_ group except for a tendency to improve at 42 days in the DMM group. This may be due to differences in the pain processing between the DMM and MIA models. The MIA model induced increased levels of pronociceptive and antinociceptive neuropeptides, whereas the DMM model was associated with more limited pain and no significant changes in spinal neuropeptide levels.^44^ Differences in the pain suppression effect of CO_2_ therapy between the MIA and DMM models might explain this, however this remains conjectural and needs further investigation. Taken together, CO_2_ therapy provides better pain and swelling control in post-traumatic OA and may have long-term therapeutic effects as a non-invasive local treatment.

OA is characterized primarily by cartilage degeneration. In the present study, OARSI grading showed numerically higher cartilage degeneration scores in both the MIA and DMM groups compared to sham; however, these differences did not reach statistical significance. Furthermore, MIA and DMM groups significantly decreased the uncalcified layer of the cartilage thickness and chondrocyte density compared to the sham group, except for chondrocyte density in the DMM group, consistent with the previous report.^45^ The CO_2_ therapy did not affect the cartilage degeneration with OA, as assessed by the OARSI score and cartilage thickness. This may be attributed to the fact that the duration of CO_2_ administration was only two weeks. A previous study reported that the therapeutic effects of CO_2_ therapy on fracture healing are duration-dependent, suggesting that a longer treatment period might be necessary to observe significant effects on cartilage in OA.^15^ Therefore, CO_2_ therapy for the cartilage morphological change may be necessary for long-term treatment. However, CO_2_ therapy significantly increased the number of chondrocytes in the uncalcified layer, which were reduced by OA induction. This means that when OA develops, cartilage and bone tissue respond to increased mechanical stress by increasing remodelling activity. In cartilage, cell proliferation and matrix synthesis increase, and there is an overall increase in the thickness of the uncalcified cartilage layer.^46,47^ Consequently, we believe that this process is accelerated by increasing the number of chondrocytes during the initial phase of CO_2_ therapy. In other words, CO_2_ transdermal absorption increases cartilage density and provides cartilage protection.

The development and pathological progression of OA are largely attributed to an imbalance between ECM synthesis and degradation. In this study, immunohistochemical results showed a decrease in type II collagen and aggrecan of MIA and DMM. Type II collagen is the most abundant protein in cartilage.^48^ It is a fibrillar collagen that acts as the structural framework of the cartilage ECM. Aggrecan is the main ECM glycoprotein of articular cartilage.^49^ Therefore, type II collagen and aggrecan expression are essential for chondrogenesis.^50,51^ In our study, the OA model demonstrated a reduced cartilage ECM that was consistent with previous studies. CO_2_ therapy tended to increase type II collagen-positive cells in the MIA (p = 0.070) and significantly increased aggrecan levels in the DMM group. This suggests that CO_2_ therapy may have prevented OA-induced reduced viability of chondrocytes and thus promoted ECM synthesis. Furthermore, our results found a significant increase in MMP13 and ADAMTS5 in the MIA and DMM groups, indicating accelerated cartilage degradation. CO_2_ therapy tended to decrease MMP13-positive cells in the MIA group (p = 0.071), and ADAMTS5 significantly decreased in the MIA and DMM groups. CO_2_ therapy may inhibit the decomposition of the cartilage matrix. In OA-induced articular cartilage destruction, the hyaluronan-aggrecan network is degraded, followed by collagen fibril degradation.^52^ MMP and ADAMTS gene family molecules play important roles in the degradation of ECM.^53,54^ ADAMTS5, an aggrecanase, plays a major role in the early stages of articular cartilage destruction, and collagenolytic MMP plays a central role in collagen degradation.^55^ In summary, our results suggest that CO_2_ therapy inhibits the development of OA by improving ECM synthesis and degradation imbalance.

Inflammation within the joints and articular cartilage plays a considerable role in OA initiation and progression.^56^ In this study, synovitis was evaluated as an index of intra-articular inflammation. Although the synovitis scores were higher in the MIA and DMM groups compared to the sham group, these differences did not reach statistical significance. CO_2_ therapy did not significantly alter synovitis scores in either model. This may be due to the short duration of just two weeks of treatment with CO_2_. Given that OA is a chronic progressive disease characterized by persistent synovial inflammation and fibrosis, a longer intervention period might be necessary to observe significant modulatory effects on synovitis.^57^ Synovial fibrosis and inflammation persistently exist in the pathological progress of OA.^58,59^ This remains conjectural and needs further investigation to determine. Thus, we believe that CO_2_ therapy inhibited the pain-like behaviour caused by inflammation, but that the treatment period was insufficient to improve synovitis within two weeks. In addition, in an inflammatory environment, the levels of pro-inflammatory cytokines such as TNF-α and IL-6 increased significantly. Among them, IL-6 inhibits the synthesis of proteoglycans and type II collagen, while TNF-α promotes their catabolism, thereby promoting the expression of matrix metalloproteinases, including MMP-13, which ultimately leads to the decomposition of ECM.^60,61^ Correspondingly, the levels of IL-6 and TNF-α were significantly increased in the MIA and DMM groups in this study (p < 0.001), except for TNF-α positive cells in the DMM group, where the increase was not significant (p = 0.053). This suggests that the cartilage was in an inflammatory state, which is consistent with previous studies.^28,45^ Our results suggest that CO_2_ therapy reduced the number of TNF-α- and IL-6-positive cells in the MIA group, as well as the number of IL-6-positive cells in the DMM group. There was no significantly reduced number of TNF-α positive cells in the DMM group, which may be due to the fact that CO_₂_ therapy demonstrated a statistically significant effect on proteoglycan and type II collagen synthesis in our model of post-traumatic OA, but not on matrix metalloproteinases. Therefore, we believe that CO_2_ therapy reduces inflammation in post-traumatic OA, mainly by reducing the inflammatory response of IL-6, which inhibits proteoglycan and type II collagen synthesis.

This study has several limitations. First, we used a small animal model. The male Wistar rats used in this study cannot fully reflect the variability found in humans (such as variations in age, race, sex, genetic variation, and lifestyle). Therefore, further clinical studies are needed to translate our results to humans. However, small animal models are preferred for preliminary screening, and the model of rats with OA appears to closely reflect the outcome in humans in terms of histopathology and function.^7^ A second limitation is that we set exposure to CO_2_ for a limited time (20 minutes per day) and duration (two weeks) in rats based on previous studies,^2,20^ and do not know whether there will be better results if the duration is longer than two weeks. We may need to determine the optimal conditions for treating OA. Additionally, all immunohistochemical analyses in this study were conducted using semiquantitative methods. Although immunohistochemical staining indicated changes in ECM-related markers such as aggrecan and type II collagen, we acknowledge that the interpretation of these results is limited by the semiquantitative nature of the method. Notably, in the DMM model, staining for type II collagen and aggrecan appeared predominantly intracellular rather than extracellular, which raises concerns about whether the signals truly reflect active ECM synthesis or instead indicate intracellular stress responses. Similar challenges have been reported in previous studies using IHC to assess matrix components in degenerating cartilage.^48,49^ Therefore, while our findings suggest a potential modulation of ECM-associated molecules by CO_2_ therapy, definitive conclusions cannot be drawn from IHC alone. Finally, we used the right and left knees as different samples. Using both joints has the advantages of minimizing the number of experimental animals needed and providing equivalent sample sizes for statistical purposes. However, its use cannot preclude chance findings attributable to intra-animal and inter-animal variation.

In conclusion, this study establishes the first preclinical evidence for transcutaneous CO_2_ therapy as a noninvasive topical application in OA, demonstrating its efficacy in attenuating disease progression through the modulation of inflammatory pathways and ECM homeostasis, particularly in mechanical load joints. While structural cartilage restoration was not achieved within the experimental timeframe, the transcutaneous CO_₂_ therapy to enhance chondrocyte density and suppress catabolic activity underscores its potential as a novel adjunctive approach for OA management. This investigation advances our understanding of CO_2_ application, proposes novel strategies for developing noninvasive treatments for OA, and provides new insights into the treatment of OA patients and other arthropathies.

## Data Availability

The data that support the findings for this study are available to other researchers from the corresponding author upon reasonable request.
